# Fabrication of electrospun poly(d,l lactide-co-glycolide)80/20 scaffolds loaded with diclofenac sodium for tissue engineering

**DOI:** 10.1186/s40001-015-0145-1

**Published:** 2015-06-05

**Authors:** Lila Nikkola, Tatjana Morton, Elizabeth R. Balmayor, Hanna Jukola, Ali Harlin, Heinz Redl, Martijn van Griensven, Nureddin Ashammakhi

**Affiliations:** Department of Biomedical Engineering, Tampere University of Technology, Tampere, Finland; AUVA Research Center, Austrian Cluster for Tissue Regeneration, Ludwig Boltzmann Institute for Experimental and Clinical Traumatology, Vienna, Austria; Department of Experimental Trauma Surgery, Klinikum rechts der Isar, Technical University Munich, Ismaninger Strasse 22, D-81675 Munich, Germany; Institute of Fiber Material Science, Tampere University of Technology, Tampere, Finland; Institute of Science and Technology in Medicine, Keele University, Staffordshire, UK

**Keywords:** Electrospinning, Poly(d,l lactide-co-glycolide), Scaffold, Drug release, Diclofenac sodium, Biodegradable

## Abstract

**Background:**

Adaptation of nanotechnology into materials science has also advanced tissue engineering research. Tissues are basically composed of nanoscale structures hence making nanofibrous materials closely resemble natural fibers. Adding a drug release function to such material may further advance their use in tissue repair.

**Methods:**

In the current study, bioabsorbable poly(d,l lactide-co-glycolide)80/20 (PDLGA80/20) was dissolved in a mixture of acetone/dimethylformamide. Twenty percent of diclofenac sodium was added to the solution. Nanofibers were manufactured using electrospinning. The morphology of the obtained scaffolds was analyzed by scanning electron microscopy (SEM). The release of the diclofenac sodium was assessed by UV/Vis spectroscopy. Mouse fibroblasts (MC3T3) were seeded on the scaffolds, and the cell attachment was evaluated with fluorescent microscopy.

**Results:**

The thickness of electrospun nanomats was about 1 mm. SEM analysis showed that polymeric nanofibers containing drug particles formed very interconnected porous nanostructures. The average diameter of the nanofibers was 500 nm. Drug release was measured by means of UV/Vis spectroscopy. After a high start peak, the release rate decreased considerably during 11 days and lasted about 60 days. During the evaluation of the release kinetics, a material degradation process was observed. MC3T3 cells attached to the diclofenac sodium-loaded scaffold.

**Conclusions:**

The nanofibrous porous structure made of PDLGA polymer loaded with diclofenac sodium is feasible to develop, and it may help to improve biomaterial properties for controlled tissue repair and regeneration.

## Background

Recently, electrospinning has been widely studied as a method for manufacturing biomimetic nanofibrous structures to be used as scaffolds for cells to grow in and proliferate [1]. Fibers in extracellular matrix (ECM) are in the nanoscale. Thus, nanofibrous structures are thought to mimic the fibrous structure of ECM and comprise good candidates for tissue engineering. Generally, the range of diameter of electrospun fibers is 5 nm to several micrometers. To date, nanofiber-based scaffolds have been manufactured from several synthetic and natural polymers and blends by electrospinning. Examples of synthetic polymers include poly(ε-caprolactone) (PCL) [2], poly(d,l-lactide) (PDLLA) [3], poly(glycolide) (PGA) [4], and their blends such as poly(d,l-lactide-co-glycolide) (PDLGA) [5] and poly(l-lactide-co-ε-caprolactone) (PDLCLA) [6, 7]. Natural polymers such as fibrin [8], collagen [9], starch [10], and chitosan [11] have also been manufactured by electrospinning in addition to blends with synthetic polymers such as collagen-PGA [12] and starch-PCL [13].

More recently, multifunctional electrospun scaffolds have been developed that contain bioactive substances [7, 14, 15]. Drug-releasing scaffolds have several advantages besides resembling ECM structure. For example, it can reduce systemic side effects caused by drugs administered systematically. Additionally, a therapeutic concentration of drugs can be reached locally. Short drug release periods have been reported from nanofiber-based scaffolds. For instance, Kim et al. studied the release of the antibiotic cefoxitin sodium from electrospun PDLGA nanofibrous scaffolds for 6 days [16]. The authors found that the maximal dosage of the drug was released during the first hour of incubation. This can be expected at least with low molecular weight polymers, which are usually used for electrospinning. In electrospinning, the polymer solution should have adequate viscosity to be able to flow through a needle, in order to form the so-called “Taylor cone,” and eventually establish nano- or microfibers onto a collector. However, when long-term release is desired, these electrospun matrices are problematic.

The aim of the current study was to develop a biodegradable nanofiber-based scaffold with prolonged drug release. Hence, in the current study, the idea was to use a high molecular weight (Mw) biodegradable polymer as a matrix. As a result of the high Mw, the degradation is expected to be slower (compared to its low Mw equivalent). Thereby, it prolongs the release of the entrapped drug. The high molecular weight polymer is, however, a challenge to an electrospinning process due to the high viscosity of the polymer solution. In the current study, the selected matrix polymer was poly(d,l-lactide-co-glycolide)80/20 (PDLGA80/20) with a high inherent viscosity (IV = 5.7 dl/g). PDLGA80/20 is a well-known synthetic biodegradable polymer, which has been used in many applications. The active agent was diclofenac sodium (DS) due to its long history as a well-known anti-inflammatory drug. Thus, the DS-releasing scaffold was prepared by electrospinning, and the drug release, microstructure, and preliminary cell tests were performed.

## Methods

### Preparation of the PDLGA80/20 scaffolds loaded with diclofenac sodium

Bioabsorbable poly(d,l lactic-co-glycolic acid) 80/20 (Purac Biochem, Gorinchem, The Netherlands) was dissolved in acetone/dimethylformamide (A/DMF, Sigma-Aldrich, Espoo, Finland) (ratio 7:1) to create a 6-wt.% solution. To load the scaffold with the drug, approximately 20 wt.% of DS (Sigma-Aldrich, Espoo, Finland) was added to the solution. PDLGA80/20 solution containing DS and plain PDLGA80/20 solutions were homogenously mixed with a magnetic stirrer. The scaffolds were manufactured by electrospinning. The electrospinning apparatus consisted of a glass spinneret having a metal needle as a capillary, a copper collector plate covered with aluminum foil, and a voltage source (Chargemaster BP 50, Simco, Hatfield, PA, USA). The system was placed in a fume chamber. The voltage source was attached to both ends so that the needle was charged positively and the collector plate grounded to zero potential. The distance between the needle tip and the sample collector was about 10 cm and the applied voltage was 20 kV. The needle was positioned about 45° downwards (measured against the surface of the fume hood) to increase the flow of polymer solution through the needle. After the electrostatic spinning process, residual solvent was removed by placing the aluminum foil into the fume chamber for 24 hours. Thus, allowing the volatile organic solvents used (i.e., acetone and DMF) to completely evaporate.

### Microstructure

The microstructure of the resulting scaffold was studied by using scanning electron microscopy (SEM). Samples were sputtered with gold by using Edwards S150 sputter coater (Edwards High Vacuum International, Wilmington, MA, USA) and imaging was done using JEOL T100 (JEOL Ltd., Tokyo, Japan) with increasing magnitudes up to × 2000. The average fiber diameter was measured from SEM micrographs (×2000) by dividing the picture to four frames and measuring diameters of 20 fibers from each frame with the aid of Image J 1.33u (Wayne Rasband, National Institutes of Health, Washington, USA). Based on these measurements, average fiber and bead diameters were calculated.

### Drug release

Drug release measurements were performed by using a UV/Vis-spectrophotometer (UNICAM UV 540, Thermo Spectronic, Cambridge, UK). Five replicate samples were precisely weighed to be 50 mg and then placed into vials filled with 5 ml of phosphate buffered solution (PBS, pH 7.4, Sigma-Aldrich, Espoo, Finland). The vials were kept in a rotating (100 rpm) incubator (Multitron AJ 118 g, Infors, Bottmingen, Switzerland) at 37 °C. For every measurement, five parallel samples were collected at suitable intervals. During the first day, samples were collected every 6 hours. Subsequently, sample collection was performed once per day. Based on the results of the measurements for the earlier points in time, later samples were collected less frequently (i.e., once per week). The vials were emptied into test tubes and then refilled with fresh buffer solution. Concentrations were measured from samples in the test tubes. PBS pH 7.4 was used for baseline calibration. The absorbance was then measured at 276 nm (*λ*_max_ for DS). The concentration of DS was calculated using a standard curve with linear regression. The formula for calculation of the concentration of DS was *y* = 0.0317× + 0.0091 for concentrations in the range of 0.5–100 μg/ml and reliability of 0.9999. After measuring the UV/Vis, the pH value was determined using a Mettler Toledo MP 225 pH (Mettler-Toledo, GmbH, Schwerzebbach, Switzerland). The drug-loaded scaffold was treated with UV light overnight to find out the effect of light treatment before cell studies. DS is known to be UV sensitive [17]. Thus, the release from UV-treated scaffold was studied in addition to non-treated scaffold.

### Cell culture studies

Cell studies were performed using mouse fibroblasts (MC3T3). The cells were purchased from European Collection of Cell Cultures (ECACC, Salisbury, UK). Before seeding the cells to unloaded and DS-loaded scaffolds, the cells were labeled with the fluorescent dye Vybrant® DiI (Invitrogen, Carlsbad, California, USA) following the manufacturer’s instructions. Briefly, 1 × 10^6^ cells were resuspended in 1-ml serum-free Dulbecco’s modified Eagle’s medium (DMEM, Sigma-Aldrich, Vienna, Austria). Subsequently, 5-μl cell-labeling solution was added to the cell suspension and incubated for 15 min at 37 °C. Next, labeled cells were washed two times with pre-warmed serum-free DMEM and were used for cell seeding experiments.

Electrospun PDLGA80/20 scaffolds were cut into 1 × 1 cm^2^ pieces and transferred to individual 24-well tissue culture plates. On the pre-wetted (i.e., with DMEM culture medium) electrospun scaffolds, 6 × 10^3^ labeled cells per cm^2^ (i.e., per scaffold) were seeded. Cell-seeded scaffolds were cultured in DMEM supplemented with 10 % fetal bovine serum (FBS, Lonza Ltd., Basel, Switzerland) and 1 % penicillin/streptomycin (P/S, Sigma-Aldrich, Vienna, Austria) for up to 7 days at 37 °C and 5 % CO_2_. One day after cell seeding, fluorescence microscope pictures were taken to evaluate the distribution of the cells in the scaffolds. Additionally, 1 and 6 days after seeding, confocal microscopy (Carl Zeiss LSM 5 PASCAL, Jena, Germany) was used for examination of attachment of the cells on the scaffolds. Pictures were analyzed by using Carl LSM Zeiss Image Browser (Carl Zeiss, Jena, Germany).

### Statistical analysis

DS release kinetic values are reported as mean ± standard deviation (*n* = 5). Statistical analysis was performed using GraphPad Prism version 6.00, (GraphPad Software, La Jolla, CA, USA). Normal distribution of the data was analyzed by applying the Shapiro-Wilk test. Student’s *t* test was used to compare DS release from PDLGA80/20 scaffolds UV-treated with the non-treated ones. All statistical analysis was performed following the recommendations of the software used. Probabilities of *p* < 0.05 were considered as significant.

## Results

### PDLGA80/20 scaffolds

**Fig. 1:**
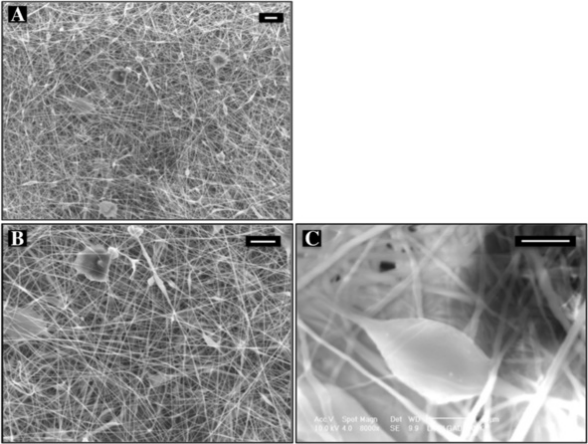
Morphological characterization of the drug-loaded nano-scaffold. Scanning electron microscopy micrographs of diclofenac sodium-loaded PDLGA80/20 electrospun nano-scaffold **a** magnification × 500, scale bar = 10 μm; **b** magnification × 1000, scale bar = 10 μm; **c** beads formation due to the drug encapsulation into the nano-fibers, magnification × 8000, scale bar = 1 μm

The thickness of the electrospun scaffolds was about 1 mm. Under low magnification SEM, polymeric nanofibers containing drug particles formed a highly interconnected porous structure (Fig. 1a). Using higher magnifications, it was seen that the structure also contained beads with different shapes (Fig. 1b, c). The average diameter of nanofibers on the drug-loaded scaffold was 949 ± 500 nm and that of the beads 17.6 ± 2.7 μm. The fiber diameter of the unloaded scaffold was 1.0 ± 0.25 μm.

### Drug release

**Fig. 2:**
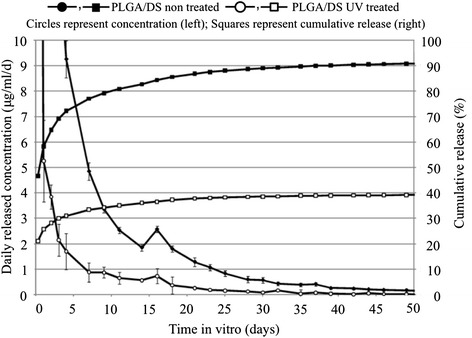
Release profiles of diclofenac sodium from PDLGA80/20 nano-scaffolds. The release study was performed in PBS at 37 °C. On the left scale, the concentration released daily is presented as micrograms per milliliter per day. The *closed circles* represent non-UV-treated nano-scaffolds, while *open circles* represent the UV-treated ones. On the right scale, the cumulative release is presented as percentage. Similarly, the *closed squares* represent non-UV-treated nano-scaffolds, while *open squares* represent the UV-treated ones

The drug release profile showed a high initial peak during the first day (burst release). Afterwards, the drug release rate was decreased considerably during the next 11 days from levels of 20 μg/ml/day to a level of 5 μg/ml/day. The release period lasted for about 60 days (Fig. 2). Cumulative release calculations showed that almost all loaded DS was released (e.g., 93 % DS was released after 50 days for the PDLGA80/20 scaffolds not treated with UV). During the drug release studies, the size of the scaffolds was notably reduced (based on visual inspection). Three and a half months after initial incubation in PBS, the scaffolds vanished completely, indicating an intermediate degradation rate. The UV treatment significantly influenced the release of the drug. A lower amount of DS was released from the UV-treated scaffolds when compared to the untreated controls for each point in time and up to 40 days (Fig. 2). After 4 days in PBS at 37 °C, levels of released DS were 1.7 ± 0.6 μg/ml/day for the UV-treated scaffolds in comparison with 9.3 ± 0.8 μg/ml/day for the non-treated ones. Seven days after incubation in PBS, the effect of the UV treatment over the DS release was less pronounced. Nevertheless, this effect was still significant (0.9 ± 0.4 μg/ml/day for the UV-treated scaffolds in comparison with 4.7 ± 0.2 μg/ml/day for the non-treated ones, *p* < 0.05). In terms of cumulative release, this represents 34 % of DS released after 7 days of incubation for the UV-treated scaffolds in comparison to 78 % of DS released for the non-treated ones.

### Cell studies

**Fig. 3:**
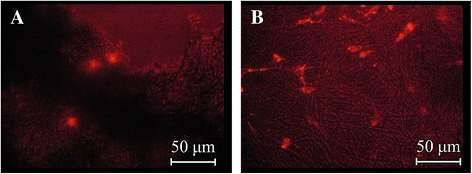
Mouse fibroblasts cells seeded onto the nano-scaffolds. Fluorescence microscopy images of mouse fibroblasts cells (MC3T3) 1 day after seeding onto the PDLGA80/20 scaffolds **a** diclofenac sodium-loaded scaffold, **b** unloaded scaffolds. Cells were previously labeled with Vybrant Dil (red fluorescence)

**Fig. 4:**
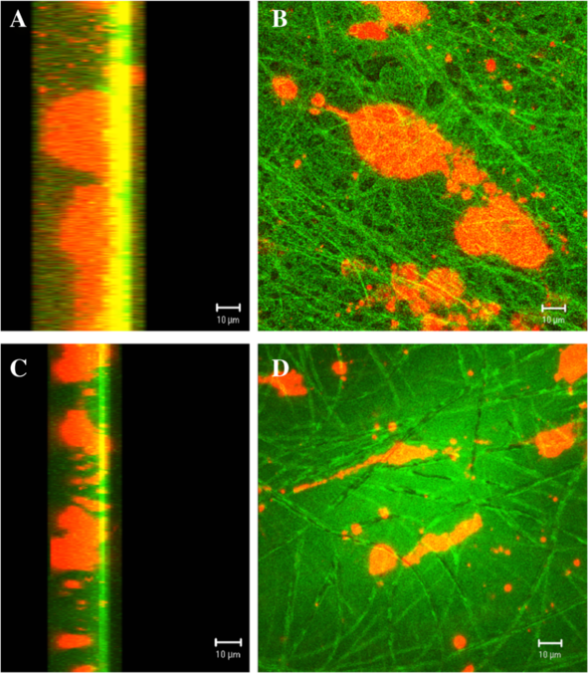
Cell attachment to the nano-scaffold. Confocal microscopy images revealed cell attachment of mouse fibroblasts cells (MC3T3) to the diclofenac sodium-loaded PDLGA80/20 scaffolds. The cells were labeled with Vybrant Dil fluorochrome immediately before seeding. **a** and **b** correspond to 1 day after seeding while **c** and **d** resemble the cells attached to the scaffold 6 days after seeding. **a** and **c** show the scaffold from the side

As early as 1 day after seeding, fluorescence microscopy confirmed the attachment of cells to the PDLGA80/20 scaffolds (Fig. 3). Confocal microscopy revealed cell attachment to both unloaded and DS-loaded scaffolds on day 1 and day 6 (Fig. 4). Interestingly, the cells have preference for the unloaded scaffolds, as more cells are observed per microscopic field in them (Fig. 3).

## Discussion

During the past decade, nanofibrous structures have intensively been studied as scaffold materials for tissue engineering [18]. One of the most popular techniques to produce these structures is electrospinning. Diverse fibrous structures can be manufactured by changing single parameters during the electrospinning process (e.g., voltage, needle diameter, and pressure). In our study, previously optimized electrospinning parameters were used to produce the PDLGA80/20 and DS-loaded PDLGA80/20 scaffolds [7]. In addition, material properties such as polymer molecular weight, solubility, volatility, and dielectric constant of the solvent together with the polymer concentration are of relevance for a successful electrospinning [19]. The selected polymer for our study, PDLGA80/20, as many synthetic polymers is soluble only in organic solvents. It dissolves in chloroform and also in acetone at low concentrations. Such solvents are often toxic for the cells and might also damage added active agents. On the other hand, the chosen drug, DS is stable and highly soluble in water, ethanol, DMF, and dimethyl sulfoxide (DMSO). It is also soluble in acetone (maximum 6 mg/ml). Acetone is a highly volatile solvent, commonly used as excipient in many pharmaceutical formulations [20]. Due to its high volatility, the solvent can be removed from the scaffolds by allowing overnight air-dry or vacuum-dry. Thus, the solvent used in the current study was chosen to be acetone. When PDLGA80/20 was dissolved in acetone, it resulted in an electrospraying rather than an electrospinning process. Subsequently, a mixture of acetone and DMF was used. As a result, the formation of the jet and Taylor cone previously described by Kim et al. [16] was enhanced. Special attention was given to use the minimal amount of DMF indispensable needed for the electrospinning process. This was based on the known toxicity associated to DMF. At the end of the electrospinning process, the collected nanomats were allowed to air-dry for 24 hours to remove solvent remnants.

Conductivity of polymer/drug solution was not measured. However, DMF has a high dielectric constant. Thus, it can be expected that conductivity of the polymer solution may have increased after addition of DMF.

Adding ions to the polymer solution, e.g., in the form of drugs, can enhance the process of electrospinning [21]. Indeed, the presence of DS enhanced the scaffold fabrication in our study. No differences between fiber diameters of unloaded and DS-loaded scaffolds were observed. Both scaffolds were prepared with the same solute concentration and under the same processing parameters. The examination of the microstructure revealed nanofibrous structures and beads. Fiber diameters varied considerably in both scaffold types, as indicated by a high standard deviation. The shape of the beads was irregular, and most of them were large when compared to the nanofibers. Another important property to be considered is the polymer molecular weight. Most ester-based biodegradable electrospun scaffolds have been made using low molecular weight (60 000–80 000 g/mol) polymers [16, 22] since they are more suitable for processing. However, low molecular weight ester-based biodegradable polymers degrade and release encapsulated agents faster than polyesters with high molecular weight. The high inherent molecular weight of the PDLGA80/20 polymer was thought to prolong the release period since the degradation is slower compared to low molecular weight PDLGA.

The main problem in prolonging drug release from nanofibrous structures lies in the nanoscale itself and in the biodegradation properties of the material. On the one hand, nanostructures are characterized by a large surface-area-to-volume ratio that is prone to hydrolysis. Viitanen et al. observed a faster release on nanostructures when compared to bulkier constructs that were manufactured using the same polymer and drug by, for instance, melt extrusion [23]. On the other hand, fast degrading biodegradable polymers release the encapsulated agent quite quickly as the material degrades. In our study, one way to attempt to control the release of DS was to use the high molecular weight PDLGA80/20 polymer. The high molecular weight is expected to decrease the degradation rate of the scaffold and thus to control the release of encapsulated DS. In addition, DS is a hydrophilic compound. Knowing that PDLGA80/20 is hydrophobic, the release rate could also be decreased since the diffusion of DS to the surroundings would be compromised.

In the current study, a concentration of 0.12 μg/ml was considered to be the lowest therapeutic concentration of DS. It is reported to be the lowest level in synovial fluid after 12 hours of oral administration of 75 mg DS which has a therapeutic effect [24]. The release rate of DS from the PDLGA80/20 scaffolds was not constant. A burst release was observed during the first day in vitro, decreasing thereafter to levels of lower than 1 μg/ml after 60 days. This might already be advantageous when controlling the inflammatory reaction after implantation. The matrix polymer also has an effect on the drug-release rate. Nikkola et al. [7] reported that the release period of drug-loaded nanofibrous poly(lactide-co-ε-caprolactone (PCL95/5) was about 90 days. PCL95/5 (molecular weight 81,500 g/mol) is a slowly degrading polymer, and thus the total release time is long. Kim et al. [16] reported drug release from PDLGA within few hours. The molecular weight in this case was 75,000 g/mol.

Considering nanofiber-based scaffolds as constructs for tissue engineering, multiple studies with cells have been made [4, 13, 18]. Cell studies of loaded scaffolds have mainly concentrated on DNA-loaded scaffolds for tissue engineering [25] and treatment of cancerous cells [26]. A few studies like the one by Chua et al. [27] reported the development of 3-methylcholanthrene-loaded dual functional scaffold seeded with hepatocytes. In the current study, the objective of cell culture tests was to evaluate the effect of DS release to the cells. It was noticed that after 1 day of culture, mouse fibroblasts attached rather to an unloaded PDLGA80/20 scaffold than to a DS-loaded scaffold. This could be explained considering both the porosity of the scaffolds and the effect of DS on the cells. DS-loaded scaffolds were characterized by denser structures with fibers closer to each other as seen in the SEM images. Thus, it can be expected that the cells may be unable to penetrate to the interior of the scaffolds. In addition, DS is known to reduce proliferation of fibroblast in vitro. Sun et al. reported that DS among other non-steroidal anti-inflammatory drugs significantly inhibited human Tenon’s capsule fibroblast growth in vitro [28]. Six days after seeding, cells attached to the DS-loaded scaffolds. An increased number of cells were observed compared to the first day after seeding. Over 70 % of entrapped DS was released during the first 6 days. Regarding sterilization methods for the scaffold, UV light has often been used due to its easy accessibility and low costs. In our study, UV light showed to have an effect on the release of DS. The retardation on the release of the drug could be due to a partial degradation of the DS. Hassanzadeh-Khayyat et al. reported that UV irradiation might partially affect the activity of DS. As a consequence, the absorbance at 276 nm (*λ*_max_ for DS) diminished or no peak at all was observed [29]. In addition, a change in coloration (to yellowish) of the DS-loaded materials after UV exposure associated with the formation of degradation products has been described [29]. This was not observed in our DS-loaded PDLGA80/20 scaffold at any point of the study. Other sterilization methods, such as gamma irradiation could be of benefit for DS-loaded constructs. Further cell culture studies as well as in vivo studies need to be performed for the developed DS-loaded PDLGA80/20 scaffold. Other therapeutic molecules, such as growth factors, could be encapsulated.

In previous studies, we have reported the fabrication of electrospun nanofiber-based scaffold using PCL95/5 [7], poly(maleic anhydride-alt-2-methoxyethyl vinyl ether) (PAM14) [30], and several poly(3-hydroxybutyrate-co-3-hydroxyvalerate) (PHBV) blends [31]. Both PCL95/5 and PAM14 were loaded with DS during the electrospinning process. In this study, we report the fabrication of PDLGA80/20 electrospun nanofiber-based scaffold loaded with DS. The present study applied previously optimized experimental conditions to fabricate a new type of electrospun nanofiber-based scaffold. When comparing the three different types of scaffolds loaded with DS, the fastest release of DS was obtained with PAM14. Both PCL95/5 and PDLGA80/20 provided a prolonged released of the DS. PCL95/5 will degrade slower than PDLGA80/20. Thus, PCL95/5 could be used in cases were long-term scaffold support is needed for the cells.

## Conclusions

Electrospinning proved to be a feasible technique for the production of DS-loaded PDLGA80/20 scaffolds. The release of DS was prolonged in vitro when compared to other drug-loaded PDLGA scaffolds, by using high molecular weight PDLGA. The DS-loaded scaffolds had small pore sizes. This may result in a limited cell colonization of the entire scaffold. Nevertheless, the porosity could be improved by adjusting the processing parameters. The DS did not present any structural changes, thus suggesting electrospinning is a suitable process for manufacturing DS-loaded scaffolds. However, in this study, we did not perform a bioactivity test on the released DS. This measurement should be considered in further studies with this system to prove that the electrospinning process did not compromise the anti-inflammatory activity of the entrapped drug. Nevertheless, we feel that we could show a successful fabrication of DS-loaded nanofiber-based scaffolds that may have a role in tissue engineering. DS is a commonly used anti-inflammatory drug. Tissue damage may appear, for example, as result of biomaterial implantation to a bone defect where the surrounding tissue may present an inflammatory process. Here, our DS-loaded scaffolds could be of good applicability. An example of an in vivo study for our construct may be to use the loaded electrospun scaffolds to cover the outside part of a bone defect. Thus, the loaded nanomats could mimic the function of the periosteum providing enclosure of the bone defect and controlling the associated inflammation.

## Abbreviations

ECM: extracellular matrix
PCL: poly(ε-caprolactone)
PDLLA: poly(d,l-lactide)
PGA: poly(glycolide)
PDLGA: poly(d,l-lactide-co-glycolide)
PDLCLA: poly(l-lactide-co-ε-caprolactone)
PDLGA80/20: poly(d,l-lactide-co-glycolide)80/20
I.V.: inherent viscosity
DS: diclofenac sodium
DMF: dimethylformamide
DMSO: dimethyl sulfoxide
SEM: scanning electron microscopy
EDS: energy dispersive X-ray
PBS: phosphate buffered solution
DMEM: Dulbecco’s modified Eagle’s medium
FBS: fetal bovine serum
P/S: penicillin/streptomycin
